# Discovery of a Recombinant Human Monoclonal Immunoglobulin G Antibody Against α-Latrotoxin From the Mediterranean Black Widow Spider (*Latrodectus tredecimguttatus*)

**DOI:** 10.3389/fimmu.2020.587825

**Published:** 2020-11-12

**Authors:** Sofie Føns, Line Ledsgaard, Maxim V. Nikolaev, Alexander A. Vassilevski, Christoffer V. Sørensen, Manon K. Chevalier, Michael Fiebig, Andreas H. Laustsen

**Affiliations:** ^1^ Department of Biotechnology and Biomedicine, Technical University of Denmark, Kongens Lyngby, Denmark; ^2^ Sechenov Institute of Evolutionary Physiology and Biochemistry, Russian Academy of Sciences, Saint Petersburg, Russia; ^3^ Shemyakin-Ovchinnikov Institute of Bioorganic Chemistry, Russian Academy of Sciences, Moscow, Russia; ^4^ Moscow Institute of Physics and Technology, Dolgoprudny, Russia; ^5^ Absolute Antibody Ltd, Redcar, United Kingdom

**Keywords:** ****envenoming, spider toxins, latrotoxin, monoclonal antibodies, phage display, widow spiders, *Latrodectus tredecimguttatus*, toxin neutralization

## Abstract

Widow spiders are among the few spider species worldwide that can cause serious envenoming in humans. The clinical syndrome resulting from *Latrodectus* spp. envenoming is called latrodectism and characterized by pain (local or regional) associated with diaphoresis and nonspecific systemic effects. The syndrome is caused by α-latrotoxin, a ~130 kDa neurotoxin that induces massive neurotransmitter release. Due to this function, α-latrotoxin has played a fundamental role as a tool in the study of neuroexocytosis. Nevertheless, some questions concerning its mode of action remain unresolved today. The diagnosis of latrodectism is purely clinical, combined with the patient’s history of spider bite, as no analytical assays exist to detect widow spider venom. By utilizing antibody phage display technology, we here report the discovery of the first recombinant human monoclonal immunoglobulin G antibody (TPL0020_02_G9) that binds α-latrotoxin from the Mediterranean black widow spider (*Latrodectus tredecimguttatus*) and show neutralization efficacy *ex vivo*. Such antibody can be used as an affinity reagent for research and diagnostic purposes, providing researchers with a novel tool for more sophisticated experimentation and analysis. Moreover, it may also find therapeutic application in future.

## Introduction

The sight of a spider causes fear and anxiety in a large number of people (1, 2). Despite their fearsome disposition, most spiders are not a threat to human health, and their bites only induce minor toxic effects (3). A handful of spider species do, however, possess a venomous bite that can cause severe damage to humans (3, 4). The unfortunate victim suffering from envenoming from these species may experience significant pain, systemic illness, or dermonecrosis (4). One such group of spiders are the widow spiders (*Latrodectus* spp.) that have a worldwide distribution with 32 recognized species (5, 6). The clinical syndrome resulting from envenoming from *Latrodectus* spp. is called latrodectism and is characterized by diaphoresis and gradually developing local and regional pain that last for hours to days (5). In case of systemic envenoming, which occurs in about one-third of cases, nonspecific symptoms, such as nausea, vomiting, headache, and fatigue, are common, but latrodectism is fortunately rarely life-threatening (4).

These symptoms are caused by the action of toxic proteins present in the venom of *Latrodectus* spp. Their venoms contain a high number of different toxins, among which α-latrotoxin (α-LTX) is the key toxin that specifically affects vertebrates (7, 8). The gene for α-LTX from the Mediterranean black widow spider (*L. tredecimguttatus*) encodes a precursor protein that undergoes proteolytic cleavage during venom maturation, resulting in the active α-LTX of 130 kDa that appears to be responsible for the clinical manifestations in human victims (9, 10). All *Latrodectus* species likely express a form of α-LTX with high functional similarity to the ortholog from *L. tredecimguttatus*, which is reflected by the limited variability (>90% nucleotide identity), conserved cysteine residues, similar lengths, and posttranslational processing signals of α-LTX sequences across species (11), which may explain the similarity of clinical manifestations observed from bites by various widow spiders (12).

Pharmacologically, α-LTX binds to multiple membrane proteins on presynaptic neuronal and neuroendocrine nerve terminals and induce spontaneous massive neurotransmitter release through vesicle exocytosis via both Ca^2+^-dependent and independent mechanisms (13, 14). Due to this function, α-LTX has played a fundamental role as a tool in the study of neurotransmitter release in vertebrates (15), although some questions about its mechanism of action remain unanswered (13). Currently, neurotransmitter exocytosis induced by α-LTX is believed to involve an initial step of α-LTX binding to extracellular cell surface membrane proteins, which has been studied *in vitro* using α-LTX. Three structurally distinct receptors have been identified to this date: (i) neurexin 1α, a neuronal protein with a single transmembrane domain (16, 17), (ii) latrophilin 1, also called CIRL (calcium-independent receptor of α-latrotoxin), a G protein-coupled receptor (18, 19), and (iii) protein tyrosine phosphatase σ (20). Once bound to a receptor, α-LTX oligomerization ensues, followed by membrane insertion and formation of a non-selective cation channel (21, 22), which leads to Ca^2+^ entry and vesicle exocytosis. How this release of neurotransmitters causes the clinical manifestations previously described is yet to be elucidated (4). The diagnosis of latrodectism is purely clinical, combined with the patient’s history of spider bite. No analytical assays exist to detect widow spider venom in blood, urine, or at the bite site (4, 23).

Antibodies are widely used in many diagnostic and therapeutic applications, and having a monoclonal antibody targeting α-LTX could be of utility in both the research setting as an affinity agent, when latrotoxins are employed for experimentation, and in the clinical setting when widow spider bites are suspected. Murine monoclonal antibodies against α-LTX have been produced and successfully applied in studies of the mode of action of the toxin (24–27); they are nowadays used for its affinity purification. Phage display technology has previously been used to discover recombinant human monoclonal antibodies against animal toxins. Still, to the best of our knowledge, no recombinant human monoclonal antibody has ever been reported against a spider toxin. Here, we report the discovery of the first recombinant human monoclonal immunoglobulin G (IgG) antibody (TPL0020_02_G9) that binds α-LTX and show *ex vivo* neutralization efficacy. This affinity agent may find applications in research, as well as it may be of utility in future therapeutic purposes due to its fully human, recombinant, and monoclonal nature.

## Materials and Methods

### Reagents

Lyophilized native α-LTX (>98% purity) was obtained from Alomone Labs (Jerusalem, Israel, LSP-130). 6,7-Dinitroquinoxaline-2,3-dione (DNQX, an AMPA receptor antagonist) and D(−)-2-amino-5-phosphonopentanoic acid (D-APV, an NMDA receptor antagonist) were obtained from Tocris Bioscience. All other chemicals used for preparation of the artificial cerebrospinal fluid (ACSF) and pipette solutions were obtained from Sigma-Aldrich.

### Biotinylation of Toxin

α-LTX was reconstituted in phosphate buffered saline (PBS). Biotin linked to *N*-hydroxysuccinimide (NHS) *via* a PEG_4_-linker (EZ-Link™ NHS-PEG_4_-Biotin, No-Weigh™ Format, Thermo Scientific, #21329) was added to the toxin solution at a molar ratio of 1:3 (toxin:biotin) and left to react at room temperature for 45 min. Buffer exchange columns (Vivacon 500, Sartorius, 5,000 Da Molecular Weight Cut-Off) were employed for purification of the biotinylated toxins. Protein concentration was assessed based on absorbances measured on a NanodropLite Spectrophotometer.

### Phage Display Selection and Assessment of Polyclonal Output

For phage display selection, the IONTAS phage display library λ was employed. This library is a human antibody phage display library with a clonal diversity of 1.6 × 10^10^, with antibodies in the form of single-chain variable fragments (scFvs), which was constructed from B lymphocytes collected from 43 non-immunized human donors (28).

Selections and primary screenings were performed as described previously (28, 29) with the following modifications: In short, for the selections, biotinylated α-LTX (5 µg/ml) was indirectly immobilized on streptavidin-coated (10 µg/ml) MaxiSorp vials. A deselection process utilizing streptavidin-coated Dynabeads was performed on the library before commencing the first selection round to deselect phages displaying streptavidin-recognizing scFvs. In the second and third selection round, neutravidin was used instead of streptavidin to limit further accumulation of potential streptavidin binders. Three rounds of selections were carried out, and the selected phage outputs were then evaluated for antigen binding. The evaluation was carried out similarly to the selections by testing whether the output phages, purified by sequential polyethylene glycol precipitation (28), from the second and third selection round displayed increased binding to either biotinylated α-LTX (5 µg/ml) indirectly immobilized on streptavidin-coated (10 µg/ml) MaxiSorp vials, streptavidin coated vials, or vials blocked in 3% (w/v) skimmed milk in PBS. Following binding of the phages to the immobilized antigens (α-LTX, streptavidin, or milk proteins), phages were eluted using 100 µg/ml trypsin and added to cultures of *E. coli* TG1 cells with a measured optical density of 0.5 at 600 nm and shaken at 150 rpm at 37 °C for 1 h. Then, the cultures were plated and incubated overnight at 30 °C on 2xTY medium plates supplemented with 2% glucose and 100 µg/ml ampicillin. The following day, the number of colony-forming units was determined.

### Subcloning and Primary Screening of scFvs

The scFv genes from selection round two and three were sub-cloned from the phage display vector using *NcoI* and *NotI* restriction endonuclease sites into the pSANG10-3F vector for expression of soluble scFvs (30) and transformed into *E. coli* strain BL21(DE3) (New England Biolabs). For each selection round, 267 individual scFv clones were picked, expressed in 96-well format, and scFv-containing supernatants were tested for binding to MaxiSorp plates coated with 5 µg/ml α-LTX, as previously described (29). For binding detection, a monoclonal scFv ELISA was performed, using a 1:20,000 dilution of ANTI-FLAG M2-Peroxidase (HRP) antibody (Sigma-Aldrich, #A8592) in 3% (w/v) skimmed milk in PBS and o-phenylenediamine dihydrochloride (OPD) solution (Sigma-Aldrich, #SLBP6518V) according to the manufacturer’s protocol. Upon initial screening, 39 scFvs were selected and sequenced (Eurofins Genomics sequencing service) using the S10b primer (GGCTTTGTTAGCAGCCGGATCTCA). The antibody framework and CDR regions were annotated and analyzed to identify unique clones.

### Conversion of scFv to IgG1 Format

Antibody variable domains were codon-optimized for expression in human cells and designed with *NheI* and *Aval* restriction sites at the 5’ and 3’ ends. Variable domains were synthesized and cloned into expression vectors containing the constant domain sequences of the respective human IgG1 heavy chain or human λ light chain. Following sequence verification, plasmids were prepared in sufficient quantity for transfection using Plasmid Plus purification kits (Qiagen). HEK 293 (human embryonic kidney 293) mammalian cells were passaged to the optimum stage for transient transfection. Cells were transiently transfected with heavy and light chain expression vectors and cultured for a further 6 days at 37 °C with shaking at 140 rpm. Cultures were harvested by centrifugation at 4,000 rpm and filtered through a 0.22-µm filter. The first step of purification was performed by Protein A affinity chromatography with elution using citrate at pH 3.0, followed by neutralization with 10% (v/v) 1 M Tris at pH 9.0. The antibody was exchanged into PBS at pH 7.2 using a PD10 desalting column (GE Healthcare). Antibody concentration was determined by UV spectroscopy, and the antibodies were concentrated as necessary. Antibody purity was determined by sodium dodecyl sulphate polyacrylamide gel electrophoresis (SDS-PAGE) and size exclusion chromatography (SEC). SEC was performed on a Superdex^®^ 200 5/150 GL column (Cytiva, #28-9909-45) connected to an Agilent 1100 system. The mobile phase was PBS, pH 7.2 (Gibco, #20012-019). Analysis was conducted at room temperature using a flow rate of 0.2 ml/min. 5 μl sample at 1 mg/ml was injected and peaks were detected using a UV absorption of 280 nm. Endotoxin testing was performed on a *Charles River* Endosafe^®^ nexgen-MCS™ platform according to manufacturer’s instructions using matching cartridges with sensitivity to 0.05 EU/ml.

### Monoclonal IgG ELISA

To test if the previously determined binding of the scFv was retained through reformatting to the IgG format, a monoclonal IgG ELISA was set up. A MaxiSorp plate was coated either with α-LTX (5 µg/ml) or *L. tredecimguttatus* whole venom (10 µg/ml) overnight, and the following day it was blocked with 3% (w/v) skimmed milk in PBS. Subsequently, the antibody was diluted in 3% (w/v) skimmed milk in PBS in a dilution series and added in triplicates to the plate. The dilution series was constructed from a monoclonal IgG stock of TPL0020_02_G9 with a concentration of 1 mg/ml and included the following concentrations: 2,000 ng/ml, 1,000 ng/ml, 500 ng/ml, 250 ng/ml, 125 ng/ml, 62.5 ng/ml, and 31.25 ng/ml. To test specificity of TPL0020_02_G9, the binding to wells coated with streptavidin, neutravidin, and wells blocked with 3% (w/v) skimmed milk in PBS was tested. In the controls, the highest IgG concentration (2,000 ng/ml) was used. For detection of binding, a 1:10,000 dilution of Anti-Human IgG (Fc-specific)-Peroxidase antibody (Sigma-Aldrich, #A0170) in 3% (w/v) skimmed milk in PBS was utilized along with a 3,3′,5,5′-Tetramethylbenzidine (TMB) substrate kit (Thermo Scientific, #34021). Absorbance was measured at 450 nm in a Multiskan FC Microplate Photometer (Thermo Scientific).

### Rat Brain Slice Preparation

Wistar rats (16–18 days old, males and females) were used in this study. All experiments were performed in accordance with the European Directive 2010/63/EU and were approved by the Local Bioethics Committee of the Sechenov Institute of Evolutionary Physiology and Biochemistry, Russian Academy of Sciences.

Rat brain was removed and chilled to 2–4 °C in a solution containing: 125 mM NaCl, 25 mM NaHCO_3_, 2.5 mM KCl, 0.1 mM CaCl_2_, 1.25 mM NaH_2_PO_4_, 3 mM MgCl_2_, and 10 mM D-glucose (pH 7.4). Coronal slices (250 µm thick), comprising the medial prefrontal cortex (mPFC), were made using a vibrotome (7000smz-2, Campden Instruments) and incubated in ACSF containing: 125 mM NaCl, 25 mM NaHCO_3_, 2.5 mM KCl, 2 mM CaCl_2_, 1.25 mM NaH_2_PO_4_, 1 mM MgCl_2_, and 10 mM D-glucose (pH 7.4; T = 22–24 °C, 305–308 mOsm/L). All solutions were aerated with carbogen (95% O_2_ + 5% CO_2_).

### Electrophysiological Recordings

Whole-cell patch clamp recordings were performed using an EPC-10 patch clamp amplifier (HEKA Elektronik GmbH, Germany). For recordings, slices were perfused with ACSF solution (T = 22–24 °C) at a rate of 2 ml/min. Pyramidal cells in the mPFC (L2/3) were visualized with an upright microscope (BX51WI, Olympus, Japan) equipped with differential interference contrast optics. The pyramidal cells had triangular bodies and apical dendrites and were recognized according to previously described electrophysiological criteria (31). The patch pipettes (2.5–3.5 MΩ) were made from borosilicate glass (WPI) using a p-97 puller (Sutter Instruments). The pipette solution contained: 135 mM KSO_3_CH_3_, 5 mM NaCl, 0.2 mM EGTA, 10 mM Hepes, 4 mM Mg-ATP, 0.3 mM Na-GTP, and 10 mM phosphocreatine (pH was adjusted to 7.3 with KOH, 295 mOsm/L). Access resistance was typically 15–20 MΩ and remained stable during the experiments (≤ 20% increase) for the cells included in the analysis. The signals were filtered at 10 kHz and sampled at 20 kHz. Reagents were applied to the perfusion ACSF.

α-LTX was dissolved to 0.5 mg/ml in 50% glycerol and aliquots were kept at −20 °C. 15 μl of α-LTX solution were then diluted with 275 μl of ACSF, incubated for 2 h at room temperature, diluted by ACSF to 1 nM, and applied to the slices. In preincubation experiments with IgG TPL0020_02_G9, α-LTX was first diluted with 75 μl of IgG solution (1 mg/ml) in PBS, and then with 200 μl of ACSF. The mixture was likewise incubated for 2 h at room temperature, diluted by ACSF to 1 nM α-LTX concentration, and applied to the slices.

### Data Analysis and Statistics

The offline data analyses of recorded spontaneous excitatory post-synaptic currents (spontaneous EPSCs) were performed using Clampfit 10.2 software (Molecular Devices) and Origin 9.1 (OriginLab Corp.). The experimental data are presented as means ± SD. The data were normally distributed (Shapiro-Wilk test). The statistical significance of the differences in effects of α-LTX and α-LTX in the presence of IgG was evaluated by Student’s two-tailed unpaired t-test. A value of p < 0.05 was considered statistically significant.

## Results

### Phage Display Selection and Screening of scFvs

**Figure 1:**
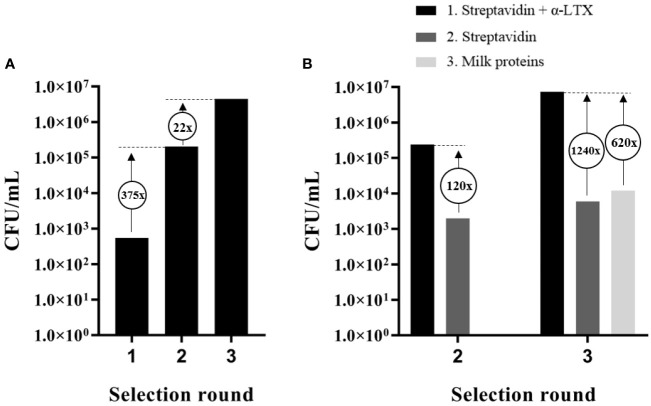
Output and assessment of phage display selections against α-LTX. **(A)** Accumulation of scFv-displaying phages from the IONTAS phage display library λ with affinity to α-LTX over three rounds of selection. An increase in CFU/ml of 375-fold and 22-fold respectively were observed between the selection rounds. **(B)** CFU/ml was determined for output phages from selection round two and three for binding to either α-LTX, streptavidin, or milk proteins. An increase of 30-fold between the second and third selection round was observed in CFU/ml for the phages that bound streptavidin-captured α-LTX. Only few phages with affinity to streptavidin or milk proteins were accumulated compared to phages with affinity to α-LTX.

**Figure 2:**
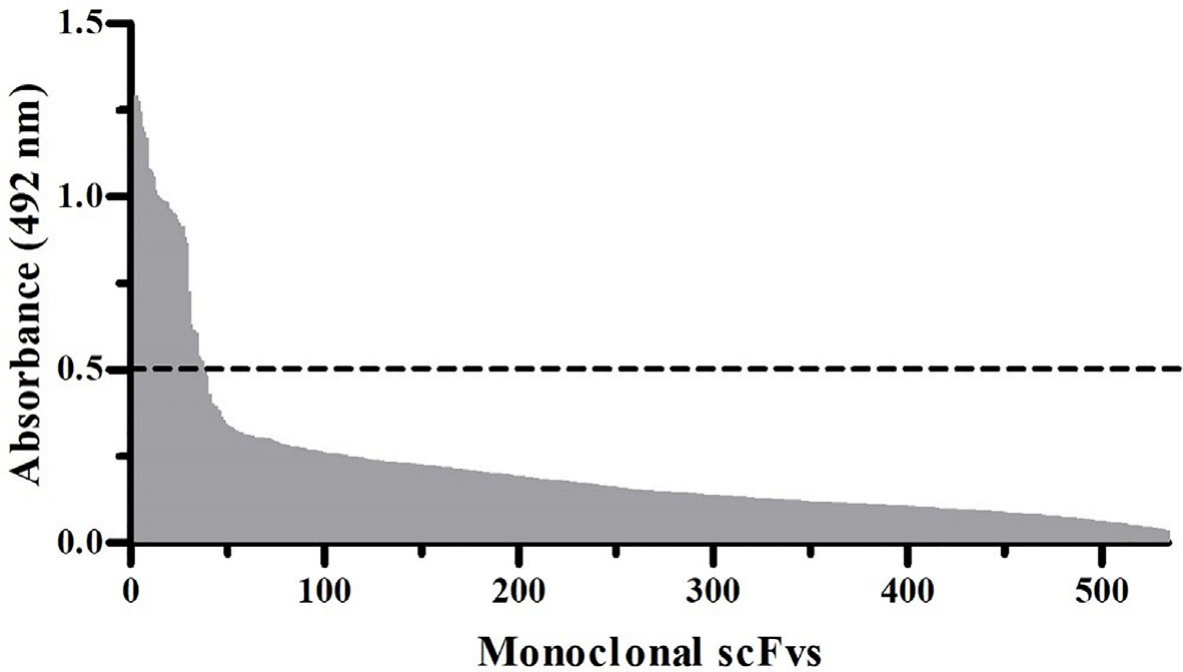
Monoclonal scFv ELISA signals against α-LTX. In total, 534 scFv clones were expressed in solution and screened for their ability to bind directly coated α-LTX. scFvs displaying a binding signal above the cut-off absorbance value of 0.5 (dotted line) at 492 nm were considered hits.

scFv-displaying phages from the IONTAS phage library λ were used to select antibody fragments against biotinylated α-LTX from *L. tredecimguttatus*. Three rounds of selections were performed to enrich the pool of phages binding the toxin (**Figure 1**). The scFv-encoding genes in the accumulated phages were isolated from both the second and third selection round, sub-cloned into the pSANG10-3F expression vector (30), 267 clones were picked from each selection round, and the scFvs were expressed in solution. Recombinant monoclonal scFvs were tested for their binding ability to directly coated α-LTX (**Figure 2**). Using a cut-off value of 0.5 in absorbance, 39 binders were selected for further characterization by DNA sequencing, yielding six unique scFvs with a distribution of 31 binders comprising one scFv (named TPL0020_02_G9), four binders comprising another scFv, and four unique binders.

### Conversion From scFv to IgG1 Format and Characterization by Monoclonal ELISA

**Figure 3:**
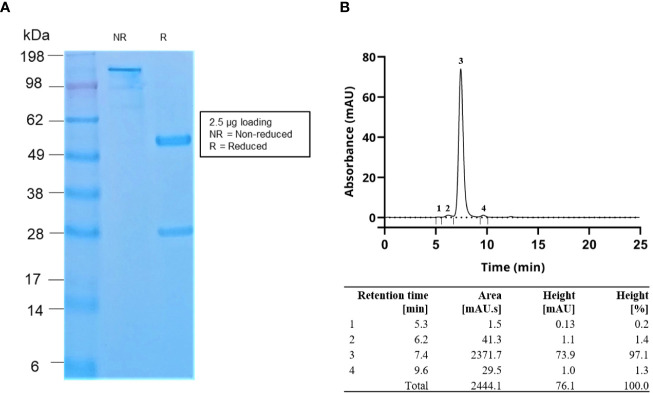
**(A)** SDS-PAGE gel image and **(B)** SEC data of the purified IgG antibodies. The SEC data demonstrated that the antibody purity was >97%. Flow rate in SEC was 0.2 ml/min.

**Figure 4:**
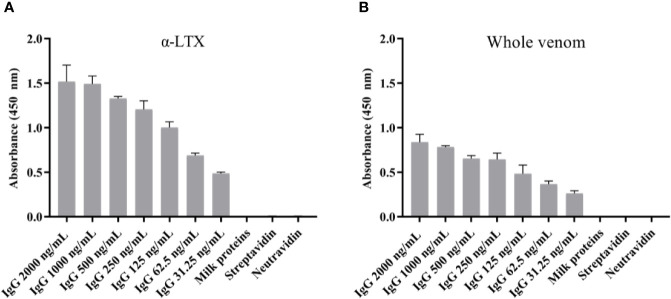
Monoclonal IgG ELISA signals against **(A)** α-LTX and **(B)** *L. tredecimguttatus* whole venom. The binding capability of TPL0020_02_G9 to α-LTX was assessed using different concentrations of IgG. The binding specificity was evaluated by testing the binding of the IgG to three controls (binding to milk proteins, streptavidin, and neutravidin) using the highest IgG concentration (2,000 ng/ml). Each column represents the average of triplicate measurements with error bars indicating the standard deviation.

The scFv that yielded the highest binding signal in the monoclonal scFv ELISA, TPL0020_02_G9, was selected for conversion to IgG1 format. Following expression by transient transfection in HEK 293 cells, the antibodies were purified by affinity protein A chromatography. The purity of the IgG was determined by SDS-PAGE and SEC, which showed >97% purity (**Figure 3**). The endotoxin level was determined as < 0.05 EU/mg by LAL chromogenic endotoxin assay. An ELISA was performed to confirm the retention of binding of the IgG, named TPL0020_02_G9, to α-LTX (**Figure 4A**). The ELISA confirmed that the IgG was able to bind α-LTX, even at the lowest dilution of 1:32,000 (31.25 ng/ml), indicating further titration might be possible. The specificity of TPL0020_02_G9 was confirmed, as control experiments showed no binding to neutravidin, streptavidin, or milk proteins at the highest IgG concentration of 1:500 (2,000 ng/ml). TPL0020_02_G9 was furthermore shown to retain binding signals when the purified α-LTX coating was exchanged for *L. tredecimguttatus* whole venom (**Figure 4B**), although, unsurprisingly given the low percentage of α-LTX in whole venom (32), the signal decreased.

### Functional *Ex Vivo* Analysis of α-LTX and its Activity Neutralized by the Discovered IgG

**Figure 5:**
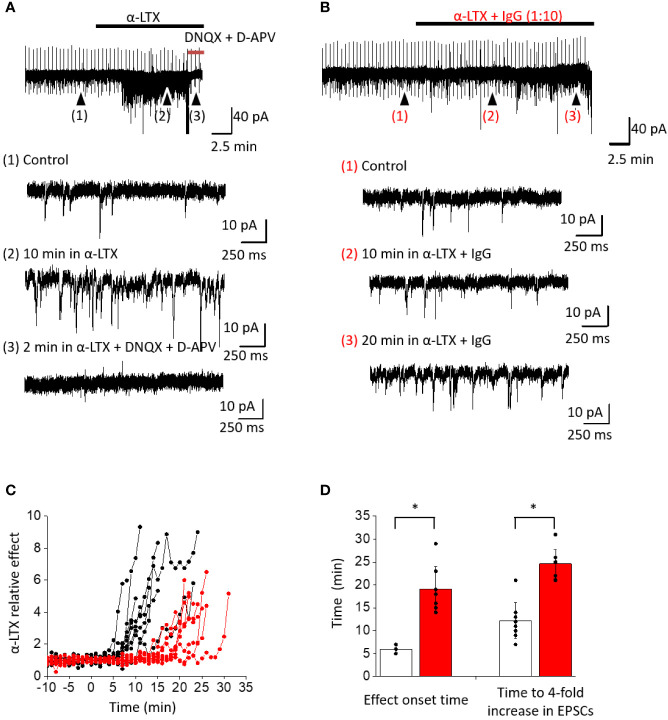
Effect of α-LTX and its mixture with IgG TPL0020_02_G9 at 1:10 ratio (w/w) on spontaneous EPSC frequency in pyramidal neurons from mPFC. **(A)** Representative recording of α-LTX action. Time course of the experiment and expanded parts in control (1), after 10 min of α-LTX application (2), and after 10 μM DNQX and 100 μM D-APV application (3) are shown. α-LTX causes a strong increase of spontaneous EPSC frequency, and selective antagonists of ionotropic glutamate receptors abolish this effect. **(B)** Representative recording of α-LTX action in the presence of IgG TPL0020_02_G9. Strong frequency increase is seen only after 20 min of application. **(C)** Time development of α-LTX effect and its modulation by IgG. Spontaneous EPSC frequencies are normalized to the average control values. The development of effect is strongly delayed in the presence of IgG (red traces) compared to control with α-LTX alone (black traces). **(D)** Characteristic times when the 3 × SD threshold is reached or spontaneous EPSC frequency is increased four-fold for α-LTX alone (white bars) and in the presence of the IgG (red bars). The differences are significant (p < 0.05, unpaired t-test).

We studied the effects of IgG TPL0020_02_G9 on the capability of α-LTX to enhance the frequency of spontaneous EPSCs recorded from brain neurons. We performed whole-cell voltage clamp recordings from pyramidal cells in the mPFC of rat brain slices. The neurons were voltage clamped at −80 mV. At this voltage spontaneous inward synaptic currents of 12.1 ± 1.6 pA amplitude were recorded, and the average frequency was 3.3 ± 1.2 Hz (n = 17) in the control (**Figure 5A**). In the presence of 1 nM α-LTX, an increase of the frequency of spontaneous EPSCs was observed (**Figure 5B**). The threshold for α-LTX effect was set as three times the frequency SD value of the control. On average, 5.8 ± 0.8 min (n = 9) were required to reach this threshold level (**Figure 5D**). The effect of α-LTX developed sharply and after 12.2 ± 3.9 min (n = 9) the spontaneous EPSC frequency exceeded its control value by more than 4 times (**Figures 5A, C, D**). In most cases, further increase in the EPSC frequency prevented its correct calculation due to a strong overlap between individual spontaneous EPSCs (**Figure–5A**). The spontaneous EPSCs were completely abolished by the application of 10 µM DNQX and 100 μM D-APV (**Figure 5A**), indicating that those EPSCs were mediated by ionotropic glutamate receptors. Our data on α-LTX action are in good agreement with previous results on α-LTX action [e.g., (33)].

Preincubation of α-LTX with IgG TPL0020_02_G9 at 1:10 ratio (w/w) did not completely inhibit the action of the toxin but markedly slowed down the development of its effect (**Figure 5**). The onset time of the effect (exceeding the 3 × SD threshold) increased to 19.1 ± 4.8 min (n = 8). Further frequency increase was almost as sharp as in control experiments without the antibody. A four-fold spontaneous EPSC frequency increase in the control took place after 12.2 ± 3.9 min (n = 9); in the presence of the IgG, this time increased to 24.6 ± 3.1 min (n = 8; **Figures 5B–D**). Based on these observations, we conclude that the IgG significantly delays the toxic effects of α-LTX.

## Discussion

Here, we report the discovery of the first recombinant human monoclonal IgG antibody against a spider toxin, which showed neutralization efficacy *ex vivo*. Due to its human, recombinant, and monoclonal nature, this antibody could potentially find utility as a therapeutic, but might also find application as an affinity reagent for research or diagnostic purposes (23). By utilizing antibody phage display, we were able to discover six unique human scFvs recognizing α-LTX from *L. tredecimguttatus.* The most promising of these six scFvs was converted to a fully human IgG1 with the purpose of increasing stability and to have an Fc fragment to increase its (therapeutic) utility. The IgG TPL0020_02_G9 was confirmed in ELISA to have retained the specificity and strong binding for α-LTX of the scFv and was also shown to retain binding when tested against *L. tredecimguttatus* whole venom. Importantly, we have found that the IgG is capable of decreasing α-LTX activity as tested in rat brain neurons, thereby demonstrating its ability to neutralize α-LTX *ex vivo*.

TPL0020_02_G9 is a recombinant human monoclonal IgG1 antibody, which possesses several advantages compared to existing animal-derived polyclonal or monoclonal antibodies. In regards to monoclonal antibodies, only antibodies of murine origin derived using hybridoma technology or phage display technology have been reported for α-LTX (24–27, 34, 35). However, due to their heterologous nature, such antibodies are not optimally suited to be developed into therapeutics for human recipients. Moreover, as a result of the recombinant origin of TPL0020_02_G9, continuous production of this antibody is independent of laboratory animals as well as spider venom. This is particularly beneficial, as venom collection is a very labor-intensive process, which requires “milking” of a large number of spiders as each spider only produces small amounts of venom (36). Most importantly, the recombinant origin of TPL0020_02_G9 ensures reproducibility, which is often a concern with animal-derived antibodies (37). This is in line with the recent recommendation in May 2020 from the European Commission’s Joint Research Centre on non-animal-derived antibodies. This recommendation urges to stop using animals for the development and production of antibodies for research, regulatory, diagnostic, and therapeutic applications by recognizing the scientific validity of non-animal-derived antibodies (38). Non-animal-derived antibodies derived from naïve display libraries, like the one used in this study for phage display selection, facilitates the selection of antibodies for specificity and affinity (39). In addition, they can be used repeatedly for the discovery of antibodies against different antigens in contrast to the necessary new immunization scheme for animal-derived polyclonal and monoclonal antibodies. The defined sequence of TPL0020_02_G9 makes it possible to engineer the antibody into various immunoglobulin isotypes, species, and to introduce specific modifications in its sequence. Furthermore, the human IgG1 format is strategically chosen, as it is compatible with therapeutic use in humans.

The antibodies discovered in this study were selected for their ability to bind to α-LTX from *L. tredecimguttatus*. However, given the high degree of homology of α-LTX isoforms across spider species (11), with α-LTX from the Australian redback spider *Latrodectus hasselti* sharing >90% amino acid identity to α-LTX from *L. tredecimguttatus* (40), it is likely that the discovered IgG may be cross-reactive towards other α-latrotoxins, although this hypothesis has yet to be tested. If cross-reactive, the IgG may find broader application as an affinity reagent in research, as well as a potential biotherapeutic against widow spider bite envenoming in future.

## Author Contributions

Conceptualization, AL. Methodology, SF, LL, MN, AV, CS, MC, MF, and AL. Validation, SF, AV, CS, and AL. Formal analysis, SF, LL, AV, CS, and AL. Investigation, SF, LL, MN, CS, MC, MF, and AL. Resources, MN, MF, and AL. Data curation, SF, LL, MN, and CS. Writing, original draft preparation, SF and AL. Writing—review and editing, SF, LL, MN, AV, CS, MC, MF, and AL. Visualization, SF, LL, MN, CS, and AL. Supervision, AL.

## Funding

This study was supported by the Villum Foundation (grant no. 00025302). AV was supported by the Russian Science Foundation (grant no. 20-44-01015).

## Conflict of Interest

MF is a shareholder and employee of Absolute Antibody, where the antibody described in this study is commercialized.

The remaining authors declare that the research was conducted in the absence of any commercial or financial relationships that could be construed as a potential conflict of interest.
